# Nrf2-mediated therapeutic effects of dietary flavones in different diseases

**DOI:** 10.3389/fphar.2023.1240433

**Published:** 2023-09-12

**Authors:** Wenkai Huang, Yuan Zhong, Botao Gao, Bowen Zheng, Yi Liu

**Affiliations:** ^1^ Liaoning Provincial Key Laboratory of Oral Disease, School and Hospital of Stomatology, China Medical University, Shenyang, China; ^2^ Liaoning Provincial Key Laboratory of Oral Disease, Department of Orthodontics, School and Hospital of Stomatology, China Medical University, Shenyang, China

**Keywords:** flavones, Nrf2, therapeutic effects, oxidative stress, inflammation, apoptosis

## Abstract

Oxidative stress (OS) is a pathological status that occurs when the body’s balance between oxidants and antioxidant defense systems is broken, which can promote the development of many diseases. Nrf2, a redox-sensitive transcription encoded by NFE2L2, is the master regulator of phase II antioxidant enzymes and cytoprotective genes. In this context, Nrf2/ARE signaling can be a compelling target against OS-induced diseases. Recently, natural Nrf2/ARE regulators like dietary flavones have shown therapeutic potential in various acute and chronic diseases such as diabetes, neurodegenerative diseases, ischemia-reperfusion injury, and cancer. In this review, we aim to summarize nrf2-mediated protective effects of flavones in different conditions. Firstly, we retrospected the mechanisms of how flavones regulate the Nrf2/ARE pathway and introduced the mediator role Nrf2 plays in inflammation and apoptosis. Then we review the evidence that flavones modulated Nrf2/ARE pathway to prevent diseases in experimental models. Based on these literature, we found that flavones could regulate Nrf2 expression by mechanisms below: 1) dissociating the binding between Nrf2 and Keap1 via PKC-mediated Nrf2 phosphorylation and P62-mediated Keap1 autophagic degradation; 2) regulating Nrf2 nuclear translocation by various kinases like AMPK, MAPKs, Fyn; 3) decreasing Nrf2 ubiquitination and degradation via activating sirt1 and PI3K/AKT-mediated GSK3 inhibition; and 4) epigenetic alternation of Nrf2 such as demethylation at the promoter region and histone acetylation. In conclusion, flavones targeting Nrf2 can be promising therapeutic agents for various OS-related disorders. However, there is a lack of investigations on human subjects, and new drug delivery systems to improve flavones’ treatment efficiency still need to be developed.

## 1 Introduction

Reactive oxygen species (ROS) and reactive nitrogen species (RNS) are highly reactive molecules comprised of free radicals and non-radical species, which are produced by multiple chemical reactions that take place in tissues and organs (Lettieri-Barbato et al., 2022). ROS include superoxide (O2−) and hydroxyl radicals (HO) radicals, while the non-radical ones include hydrogen peroxide (H_2_O_2_). The prevalent RNS include nitric oxide (NO) and peroxynitrite (ONOO−) (Lian et al., 2022). At the physical level, these regulatory mediators play a crucial role in maintaining cell homeostasis. They are essential for transmitting important proliferative cascades and function as signal molecules (Lettieri-Barbato et al., 2022; Nebbioso et al., 2022). The body typically regulates the production and elimination of ROS/RNS to maintain balance (Lian et al., 2022; Socha et al., 2022). However, an imbalance of pro-oxidant and antioxidant molecules caused by pathological conditions can result in a stress status known as oxidative stress (OS), which inevitably causes damage to proteins, lipids, and DNA (Lian et al., 2022; Mates et al., 2012). Increasing evidence implies that OS is associated with many diseases, such as neurodegenerative diseases (Esmaeili et al., 2022), chronic metabolic diseases (Jiang et al., 2021), diabetes mellitus (Black, 2022), cardiovascular disorders (Hou et al., 2023), and cancers (Zahra et al., 2021).

Nuclear factor erythroid 2-related factor 2 (Nrf2) is a redox-sensitive transcription encoded by the gene NFE2L2, which is regarded as the master regulator of the cellular antioxidant response as it can modulate a large number of antioxidant and cytoprotective genes (Davinelli et al., 2022). Under basal conditions, Nrf2 is sequestered in the cytoplasm. It binds to its repressor Kelch Like ECH Associated Protein 1 (Keap1), which prevents it from nuclear translocation and mediates its poly-ubiquitination and proteasomal degradation (Boorman et al., 2022). When oxidative or electrophilic stress occurs, reactive cysteines within Keap1 can be modified, therefore allowing Nrf2 to avoid ubiquitination and enter the nucleus, where it binds to downstream antioxidant response elements (ARE) and induces the transcription of a series of antioxidant genes, such as heme oxygenase-1 (HO-1), NAD(P)H-quinone oxidoreductase 1 (NQO1), superoxide dismutase (SOD), glutathione reductase (GR) and catalase (CAT). Given these functions, Nrf2 can be a protective target against the development and progression of many diseases (Davinelli et al., 2022; Luan et al., 2022; Atalay Ekiner et al., 2022). However, Nrf2 has contradictive roles in cancer biology. During the early stages of carcinogenesis, transient Nrf2 induction has benefits for countering carcinogens and mutagens and has protective effects against OS-induced malignant transformation in normal cells. However, in tumorous conditions, some changes, like somatic mutations and epigenetic modifications of Keap1 and Nrf2, have been reported to lead to prolonged Nrf2 activation, which in turn, decreases the apoptotic susceptibility of cancer cells and renders them resistant to chemo-and radio-therapy (Zhang et al., 2022; Pouremamali et al., 2022).

Flavonoids are an important class of natural polyphenolic compounds that abundantly exist in plants and fruits. Consuming flavonoids found in vegetables and fruits is believed to be beneficial for human health (Hamsalakshmi et al., 2022; Caporali et al., 2022). Flavonoids can be classified into several subgroups, including flavones, flavonols, flavanones, flavanonols, flavanols (flavan-3-ols), isoflavones, and anthocyanins according to their chemical structures (Saini et al., 2022). Among the different types of flavonoids, flavones are a crucial category that has been extensively studied (Singh et al., 2014). Until now, they have exhibited numerous pharmacological impacts, such as anti-diabetic (Rahman et al., 2022), hepatoprotective(Noor et al., 2022), anti-cancer(Islam et al., 2021), cardioprotective(Khan et al., 2021), neuroprotective properties (Hamsalakshmi et al., 2022). These extraordinary therapeutical effects may come from their regulatory effects on the Nrf2 signaling pathway, which potentiate them as antioxidative, anti-inflammatory, and anti-apoptosis defenders (Kaushal et al., 2022; Pleeging et al., 2022). Orientin has been indicated to improve the antioxidant ability and attenuate D-GalN/LPS-induced liver damage in mice via activating the Nrf2/ARE pathway (Li et al., 2022a). Apigetrin oral administration activated the Nrf2 pathway and inhibited the NF-κB pathway, subsequently inhibiting inflammation and oxidative stress, thereby alleviating LPS-induced acute otitis media (Guo et al., 2019). In this review, we focus on the protective effects of flavones targeting Nrf2 in different conditions and summarize the regulation of the Nrf2 pathway.

## 2 Flavones: structure and food sources

**FIGURE 1:**
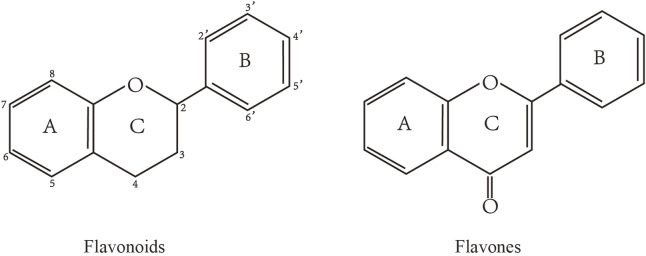
Basic structures of flavonoids and flavones.

Flavonoids are characterized by a C6-C3-C6 carbon skeleton structure containing two aromatic rings (A and B) linked by a three-carbon chain that can form an oxygenated heterocycle (ring C) with ring A (Chagas et al., 2022). There are minor structural variations among subclasses of flavonoids, including the position of the bond between ring B and ring C, degree of unsaturation, and ring C oxidation (Chagas et al., 2022). Flavones are characterized by a double bond between C2 and C3, a ketone in the position of 4 of ring C, and the attachment of B-ring at C2 (Sekaran et al., 2022). (Figure 1). Based on similar features in their fundamental structures, the number and position of the hydroxyl groups constitute a decisive factor in the biological activities of these compounds. It has been documented that the property as antioxidants of flavones primarily arises from phenolic hydroxyls attached to their framework (Singh et al., 2014; Speisky et al., 2022). Another factor deciding the bio-activity of flavones is the form they exist. Most flavonoids in edible plants are glycosylated, including O-glycosylated and C-glycosylated, which have poor lipophilicity and are ineffective at transporting across membranes. For metabolism and enhancing biological activity, glycosides must be hydrolyzed to aglycones by glycosidases in the intestine (Speisky et al., 2022). Most studies use flavones’ sugar-free form (aglycone) to investigate their pharmaceutical values (Sudhakaran and Doseff, 2020). In this review, we concentrate on common flavones in aglycone form, including apigenin, luteolin, chrysin, baicalein, wogonin, diosmetin, nobiletin, tangeretin, acacetin, and oroxylin A.

Flavones are widely present in different foods and beverages, and their typical food resources include *citus* fruits, vegetables, herbs, and grains (Sudhakaran and Doseff, 2020). Luteolin and apigenin are the main flavones in plant-based foods, such as parsley, grapes, onions, and celery. They have many biological effects, including anti-inflammatory, antioxidation, and anticarcinogenic (Chagas et al., 2022; Imran et al., 2019; Zhou et al., 2016). *Scutellaria baicalensis Georgi* is a vital component of traditional Chinese medicine, and its dried roots have a long history of treating bitterness, cold, diarrhea, hemorrhage, and inflammation. This plant from the Lamiaceae family is rich in many kinds of flavonoids and is a primary source of baicalein, wogonin, and oroxylin A (Ahmadi et al., 2022; Wang L. et al., 2022). Another group with plenty of physiological effects is the methoxylated flavones, such as tangeretin, diosmetin, and nobiletin, which are commonly found in the *citrus* family (Raza et al., 2020; Mei et al., 2022; Huang et al., 2022). The molecular formula, structure, and major sources of flavones discussed in this review are summarized in Table 1.

**TABLE 1:** Flavones in this review.

Flavones	Molecular formula	Structure	Major sources	Reference
apigenin	C_15_H_10_O_5_ 270.24 g/mol	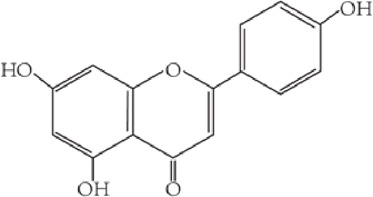	orange, parsley, onions, tea, celery, grapes and wheat sprouts	(Zhou et al., 2016)
luteolin	C_15_H_10_O_6_ 286.24 g/mol	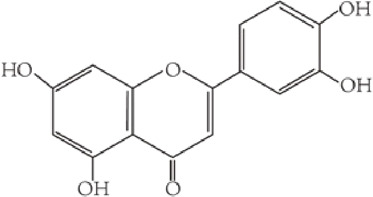	celery, chrysanthemum flowers, sweet bell peppers, carrots, onion leaves, broccoli, and parsley	(Imran et al., 2019)
baicalein	C_15_H_10_O_5_ 270.24 g/mol	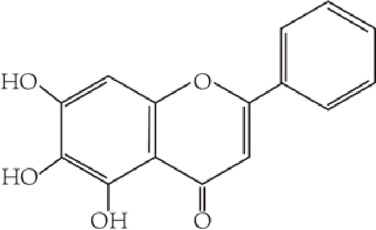	Scutellaria baicalensis Georgi	(Ahmadi et al., 2022; Wang et al., 2022a)
wogonin	C_16_H_12_O_5_ 284.26 g/mol	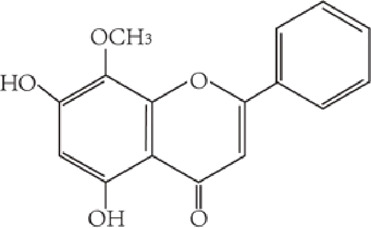	Scutellaria baicalensis Georgi	(Ahmadi et al., 2022; Wang et al., 2022a)
oroxylin A	C_16_H_12_O_5_ 284.26 g/mol	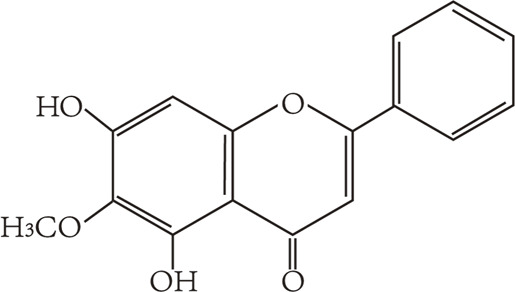	Scutellaria baicalensis Georgi et al	(Ahmadi et al., 2022; Wang et al., 2022a; Sajeev et al., 2022)
chrysin	C_15_H_10_O_4_ 254.23 g/mol	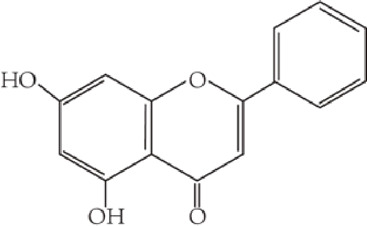	Passiflorasp, honey and propolis	(Naz et al., 2019)
acacetin	C_16_H_12_O_5_ 284.26 g/mol	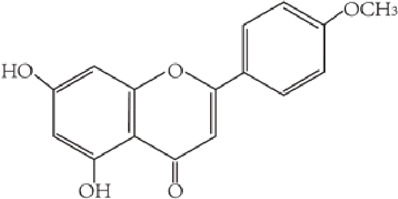	safflower, propolis, and Asteraceae plants	(Singh et al., 2020)
tangeretin	C_20_H_20_O_7_ 372.37 g/mol	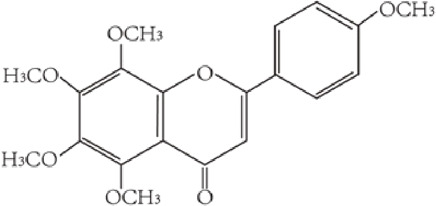	Citrus family	(Raza et al., 2020)
diosmetin	C_16_H_12_O_6_ 300.26 g/mol	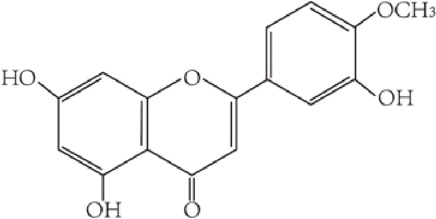	citrus fruits, legumes, olive leaves and Menthae Haplocalycis herba	(Mei et al., 2022)
nobiletin	C_21_H_22_O_8_ 402.39 g/mol	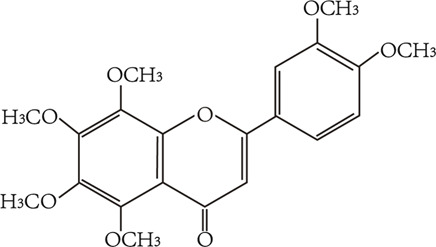	citrus peels	(Huang et al., 2022)

## 3 Regulation of Nrf2 and its association with inflammation and apoptosis

### 3.1 Structure and regulation of Nrf2

In humans, the Nrf2 protein comprises 605 amino acids and contains seven highly conserved functional domains named Nrf2-ECH homology 1 (Neh1)-Neh7 (Saha et al., 2020). The Neh1 protein contains a basic-region leucine zipper (bZIP) motif, enabling it to form dimers with small muscle aponeurosis fibromatous (sMaf) proteins. These dimers regulate the binding of Nrf2-ARE in the nucleus, leading to the activation of transcription for antioxidant enzymes (Shaw and Chattopadhyay, 2020). Neh2 contains two significant motifs, DLG and ETGE, which are necessary for the process that Nrf2 interacts with its negative regulator Keap1 (Ahmed et al., 2017). The C-terminal of Neh3 serves as a transactivation domain by binding to the transcription coactivator, CHD6. Analogously, Neh4 and Neh5 can modulate ARE-dependent genes via binding to another transcriptional co-activator cAMP response element-binding protein (CREB)-binding protein (CBP) (Nioi et al., 2005; Katoh et al., 2001). DSGIS and DSAPGS motifs in Neh6 are involved in the keap1-independent degradation of Nrf2 as they can bond to *ß*-transducin repeat-containing protein (β-TrCP) (Rada et al., 2012). Finally, the Neh7 domain suppressed the Nrf2-ARE signaling pathway through the attachment to the retinoic X receptor *a* (RXRα) (Wang et al., 2013).

The regulation of Nrf2 activation can be divided into two pathways, including the canonical (Keap1-dependent) pathway and the non-canonical (Keap1-independent) pathway (Atalay Ekiner et al., 2022). Under physiological conditions, Keap1 homodimerizes and binds to the ETGE and DLG motifs of Nrf2. Then Keap1-Nrf2 complex binds to the Cullin-based (Cul3) E3 ligase via the Neh2 domain of Nrf2 to form the Keap1-Cul3-RBX1 complex, which promotes the ubiquitination and proteasomal degradation of Nrf2 induced by the 26 S proteasome (Sivandzade et al., 2019). Under stress circumstances such as electrophiles or ROS over-generation, the cysteine residues of Keap1 undergo oxidative modification. This process allows Nrf2 to dissociate from the Keap1-Cul3-RBX1 complex and translocate to the nucleus, then Nrf2 combines with sMaf and ARE and further enhances the transcriptional activities of various antioxidant genes (Bellezza et al., 2018).

Except for the Keap1-dependent pathway, several kinases can regulate the activity of Nrf2 in a Keap1-independent manner. Glycogen Synthase Kinase-3 (GSK-3) is a serine/threonine protein kinase that has been reported to phosphorylate Nrf2 in the Neh6 domain. This process facilitates Nrf2 to be recognized by *ß*-TrCP, which targets Nrf2 for ubiquitination and proteasomal degradation (Cuadrado, 2015). It has been shown that GSK-3 can be negatively regulated by PI3K/AKT signaling through phosphorylation at Ser9 of GSK-3β or Ser21 of GSK-3α. Thus, Nrf2 activity is promoted by PI3K/AKT signaling pathway (Wu et al., 2022). Besides, the mitogen-activated protein kinase (MAPK) family members c-Jun N-terminal kinase (JNK), extracellular regulated protein kinase (ERK), and p38 MAPK (p38) have been demonstrated to modulate Nrf2 activity via phosphorylate Nrf2 at various sites (Matzinger et al., 2018). The ERK and JNK are more indicated to regulate Nrf2 activity positively; however, P38 MAPK has been suggested to both activate and inhibit Nrf2 expression (Liu et al., 2021). AMP-activated kinase (AMPK), a trimeric serine/threonine kinase, also boosts Nrf2 response in several ways, including phosphorylating the Neh1 domain to prevent Nrf2 nuclear export and phosphorylating GSK-3β (Liu et al., 2021; Petsouki et al., 2022). Similarly, Protein kinase C (PKC) phosphorylates Nrf2 at Ser-40, which is located on the Neh2 domain, thereby disturbing the interaction between Keap1 and Nrf2 to promote Nrf2 nuclear translocation (Shaw and Chattopadhyay, 2020; Huang et al., 2002). The protein Sequestosome 1 (P62) can not only compete with Nrf2 for Keap1 but also lead to autophagic degradation of Keap1 by sequestering Keap1 in autophagosomes, thereby P62 assists Nrf2 molecules to escape from Keap1-mediated ubiquitination and degradation (Liu et al., 2022). In contrast, the tyrosine kinase Fyn has been reported to phosphorylate tyrosine-568 in Nrf2, which supports Nrf2 nuclear export and degradation (Bryan et al., 2013).

**FIGURE 2:**
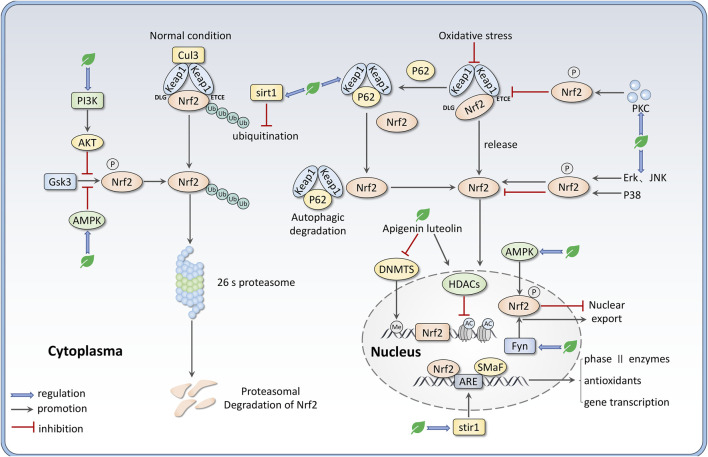
Mechanisms that flavones regulate the Nrf2 signaling pathway, including intervening the binding between Nrf2 and Keap1 via PKC-mediated Nrf2 phosphorylation and P62-mediated Keap1 autophagic degradation; regulating Nrf2 nuclear translocation by various kinases like AMPK, MAPKs, Fyn; disturbing Nrf2 ubiquitination and degradation via sirt1 and PI3K/AKT/GSK3 axis. Besides, apigenin and luteolin can regulate Nrf2 expression through epigenetic alternation such as demethylation at the promoter region and histone acetylation.

There are still many other ways for Nrf2 regulation, including transcription regulation. For example, miRNAs such as miR-144, miR-34a, and miR-28 are involved in the post-transcriptional regulation of Nrf2 (Matzinger et al., 2018; Bryan et al., 2013), although these are not main mechanisms for flavones to regulate Nrf2. Growing evidence indicates that epigenetic modifications like DNA methylation and histone modifications (methylation, acetylation, and phosphorylation) can regulate Nrf2 expression (Zhang et al., 2022). Sirtuin (SIRT) is a group of NAD + -dependent histone deacetylases that can deacetylate Nrf2 to prompt its transcription. Sirt1 has been demonstrated to enhance the activity of the Nrf2/ARE pathway through deacetylating and reducing the ubiquitination of Nrf2, decreasing Keap1 expression, and increasing ARE-binding ability (Wang et al., 2019; Patel et al., 2022). Besides, many phytochemicals can potentially prevent diseases via altering epigenetic modulation at the promoter region of Nrf2 (Thiruvengadam et al., 2021). We summarize the mechanisms of flavones regulating Nrf2 expression in Figure 2.

### 3.2 Nrf2: a modulator in inflammation and apoptosis

Interactions between OS and inflammation are firmly established in many disorders. In the inflammation process, immune cells, such as monocytes and neutrophils, are recruited to the injury sites, producing overwhelming ROS and RNS (Kaulmann and Bohn, 2014). Meanwhile, ROS and RNS accumulation promotes inflammatory response progress and mediates signal transduction. Nrf2 and NF-κB are key components cooperating to control cellular OS and inflammation (Davinelli et al., 2022). This review focuses on the relationship between Nrf2 and NF-κB signaling pathways.

The nuclear factor κB (NF-κB) family contains five members, including RelA (p65), RelB, c-Rel, p50 (NF-ĸB1), and p52 (NF-ĸB2) (Zinatizadeh et al., 2021). P65/50 dimer is considered the most common form to activate transcription (Thoma and Lightfoot, 2018). In resting conditions, NF-κB dimers are stored in the cytoplasm and inactivated by its inhibitor IκB proteins. The IκB family includes three typical members IκBα, IκBβ, and IκBε. They cover the nuclear localization sequence of NF-κB to inhibit its nuclear translocation (Socha et al., 2021). When stimuli like OS occurs, IκB kinase (IKK) gets activated to phosphorylate IkB proteins and lead them to ubiquitination and proteasomal degradation. Therefore, NF-κB can be released and translocate to nuclear to bind with DNA, which promotes the expression of proinflammatory cytokines such as Interleukin-1 (IL-1), Interleukin-6 (IL-6), tumor necrosis factor-α (TNF-α), Cyclo-oxygenase 2 (COX-2), intercellular cell adhesion molecule-1 (ICAM-1), vascular adhesion molecules, inducible nitric oxide synthase (iNOS) and others (Saha et al., 2020). Much evidence suggests that Nrf2 negatively modulates the NF-κB signaling pathway by several mechanisms. Firstly, Nrf2 activation can upregulate the expression of antioxidant genes to enhance ROS scavenging, thus inhibiting OS-induced NF-κB activation (Soares et al., 2004). Secondly, Keap1/Nrf2 pathway blocks NF-κB release from IκB proteins. Keap1 has been proven to degrade IKKβ through ubiquitination (Lee et al., 2009). Nrf2 also inhibits IκB-α proteasomal degradation, thus preventing NF-κB nuclear translocation (Krajka-Kuzniak and Baer-Dubowska, 2021). Besides, upregulation of Nrf2 induces the expression of Ho-1, which catalyzes haem to be degraded to CO, Fe2+, and converts biliverdin to bilirubin. This process is related to inhibiting NF-κB-activated pro-inflammatory factors (Wu et al., 2022). Furthermore, NF-κB activation induces the production of inflammatory mediators like COX2. 15days-PGJ2, a product derived from COX2, can inhibit NF-κB via targeting Peroxisome proliferator-activated receptor γ (PPARγ) and activate Nrf2 by reacting with Keap1 (Tastan et al., 2022). At last, Nrf2 and NF-κB inhibit each other by competing for the transcription coactivator CBP (Liu et al., 2008).

On the other hand, Nrf2 can be an essential regulator via mediating ROS, which can stimulate the three major pathways regulating apoptosis, including mitochondrial, extrinsic, and endoplasmic pathways, directly or indirectly (Redza-Dutordoir and Averill-Bates, 2016). For the mitochondrial pathway, regulation of the permeability of the inner mitochondrial membrane (IMM) is an essential process (Halestrap, 2009). The IMM is physically impermeable, but the permeability increases under excessive ROS stimulation, thus allowing protons access to the mitochondrial matrix with loss of mitochondrial transmembrane potential (MMP). Meanwhile, the mitochondrial matrix swells as water enters, leading to the rupture of the outer membrane (Halestrap, 2009; Bayir and Kagan, 2008). Increased outer mitochondrial membrane (OMM) permeabilization leads to the release of cytochrome c and subsequent assembly of the apoptosome, which is crucial for activating caspase-9. Apoptosis executioner caspases-3, -6, and -7 are furtherly activated by Caspase-9 (Crawford and Wells, 2011). In addition, ROS can activate the tumor suppressor protein p53 and c-Jun (JNK), further activate pro-apoptotic Bcl-2 proteins (Bax, Bak) translocated to OMM, and inhibit the property of anti-apoptotic Bcl-2 proteins. ROS can also cause mitochondrial DNA (mtDNA) damage, finally resulting in loss of mitochondrial membrane potential and impairment of ATP synthesis (Socha et al., 2022; Redza-Dutordoir and Averill-Bates, 2016).

**FIGURE 3:**
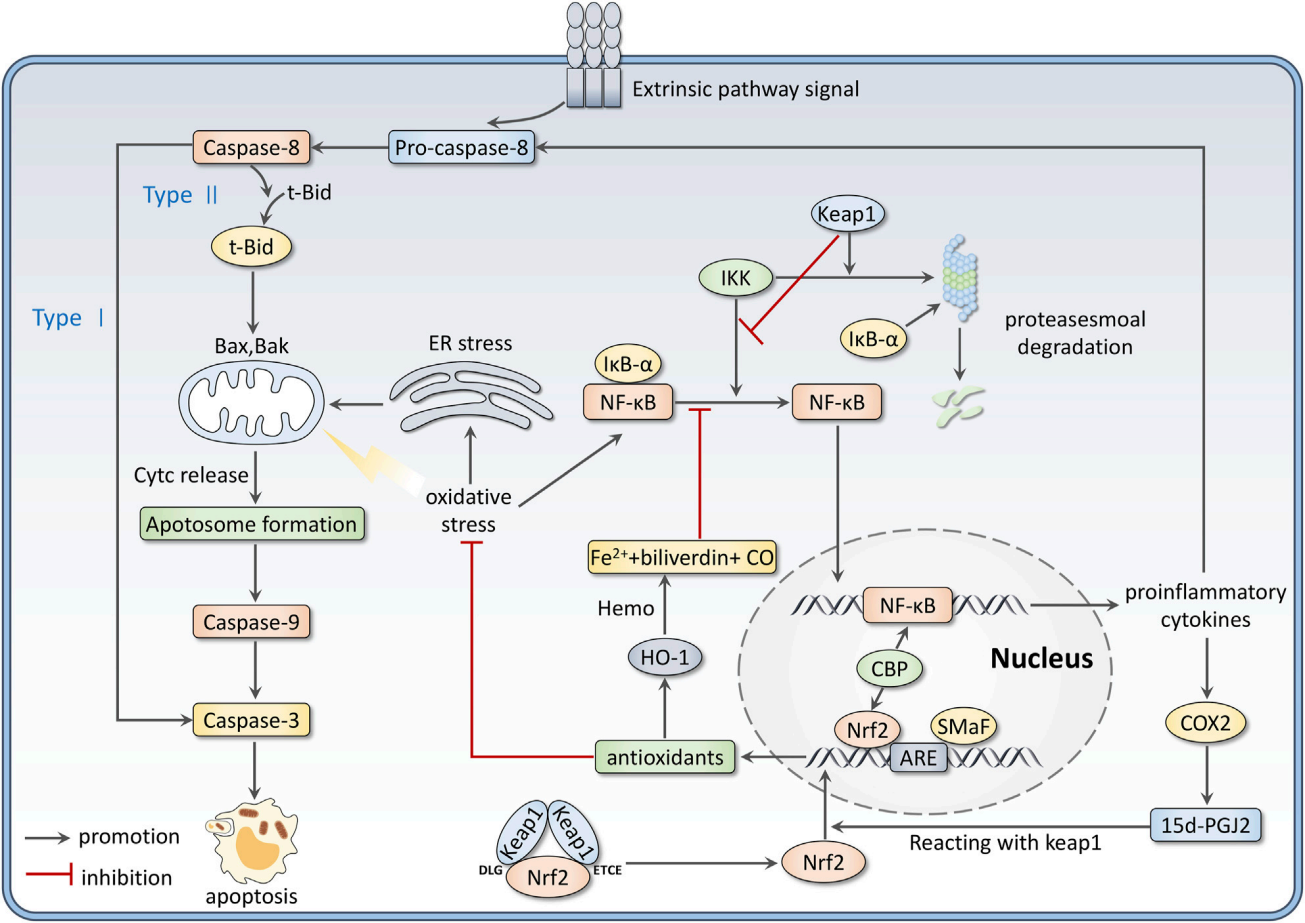
Nrf2 modulates inflammation and apoptosis. Firstly, there are interactions between the Nrf2 and NF-κB pathways. Besides, Nrf2 activation could scavenge ROS, which directly or indirectly stimulates the three ways of apoptosis.

The extrinsic pathway is mediated by receptors of extrinsic signals (such as TNF-α and Fas ligand), which can be stimulated by increased ROS (ArulJothi et al., 2022). The activation of these receptors can cause the activation of caspase-8, which mediates the execution phase of apoptosis by two mechanisms. In type1 cells, caspase-8 directly activates caspase-3; for the type2 apoptosis, activated caspase-8 cleaves pro-apoptotic Bcl-2 family protein Bid to t-Bid, the latter one transits to the mitochondria, interacting with Bax/Bak to permeabilize OMM and cause the release of apoptogenic cytochrome c (Sinha et al., 2013). In endoplasmic pathways, under the stress of excessive ROS, the endoplasmic reticulum (ER) releases more calcium ions, which can be incorporated by the mitochondria. Mitochondrial Ca^2+^ accumulation can trigger the formation of pores on the IMM, and this leads to osmotic swelling, OMM rupture, cytochrome c release, and apoptotic initiation (Burton and Jauniaux, 2011; Orrenius et al., 2015). Figure 3 summarizes the role of Nrf2 as a modulator in inflammation and apoptosis.

## 4 Protective effects of flavones targeting Nrf2

### 4.1 Neurodegenerative diseases

Neurodegenerative diseases (NDDs) are a kind of progressive, chronic, incurable disorders that could cause disability and death and significantly threaten human health. OS, inflammatory and apoptotic pathways played a pivotal role in the pathogenesis and progress of NDDs (Gravandi et al., 2021). In this context, Patel M et al. found that apigenin could prevent dyskinesia and relieve the degree of neuronal degeneration in a Parkinson’s disease (PD) model induced by LPS. Apigenin treatment dose-dependently restored Dopamine (DA) and Gamma-aminobutyric acid (GABA) levels and decreased glutamate levels in the striatum of rats. These protective effects were related to inhibiting the NF-κB signaling pathway and activating the Nrf2 pathway (Patel and Singh, 2022). Besides, apigenin and luteolin could alleviate the cytotoxicity induced by LPS and rotenone in Murine BV2 microglia cells via regulating inflammatory mediators TNF-α, IL-1β, IL-6 and promoting Nrf2 accumulation (Chen et al., 2020; Elmazoglu et al., 2020). 6-hydroxydopamine (6-OHDA) is a hydroxylated analogue of DA, an OS inducer and cytotoxic for catecholaminergic neurons. Another flavone, baicalein, effectively activated the Keap1/Nrf2/HO-1, PKCα, and PI3K/AKT signaling pathway to prevent PC12 cells from 6-OHDA-induced oxidative damage. (Zhang et al., 2012). Interestingly, chrysin combined with protocatechuic acid (PCA) diminished 6-hydroxydopamine (6-OHDA)-induced neurotoxicity in PC12 cells and blocked chemically induced dopaminergic neuron loss in zebrafish and mice. Actually, chrysin co-treated with PCA upregulated the levels of Nrf2-related proteins, reversed the loss of anti-oxidative enzyme activities, and inhibited NF-κB p65 subunit phosphorylation (Zhang et al., 2015).

Alzheimer’s disease (AD) is a neurodegenerative disease associated with multiple pathological alternations. For example, abnormal hyperphosphorylation of microtubule-associated protein Tau is related to OS, synaptic failure, and neuronal apoptosis at the early AD stage. Ni-Ni Chiang et al. demonstrated that apigenin could reduce Tau aggregation and protect cells against Tau neurotoxicity by multiple mechanisms, including rescuing the reduced HSPB1 and Nrf2 to scavenging ROS and upregulating the anti-apoptotic regulator Bcl-2 (Chiang et al., 2021). Aβ peptide fragments deposition and the consequent OS is another factor leading to neuronal impairment and AD development. Ghasemi et al. injected Aβ peptides into rats’ hippocampus to create an AD animal model. They discovered that rats administered nobiletin exhibited less cognitive impairment and lower levels of Toll-like receptor 4 (TLR4), NF-κB, and TNF-α but higher expression of Nrf2 in the hippocampus. (Ghasemi-Tarie et al., 2022).

#### 4.1.2 Ischemia-reperfusion (IR) and hemorrhagic injuries

Ischemia-reperfusion (IR) injury refers to a condition when an organ’s blood supply is obstructed and then restored, causing reoxygenation. This process leads to the accumulation of ROS, which can trigger apoptosis and inflammation (Sadrkhanloo et al., 2022). Guo et al. demonstrated that apigenin could relieve OS and suppress apoptosis of neuron-like cells after oxygen and glucose deprivation/reperfusion (OGD/R) by activating the Nrf2 pathway and inhibiting p53-related apoptotic genes (Guo et al., 2014). Yuan et al. found that pre-treating SH-SY5Y cells with baicalein alleviated the cell viability reduction, intracellular ROS accumulation, and lactate dehydrogenase (LDH) release caused by OGD, but knocking down Nrf2 reversed these effects. After conducting an *in vivo* study, it was confirmed that administering baicalein had a positive impact. The treatment helped to reduce the neurological score, decrease brain edema, and lower the infarct volume of I/R rats (Yuan et al., 2020). Similarly, recent studies found that diosmetin could exert protective effects in the middle cerebral artery occlusion (MCAO) model *in vivo*. The mechanisms included the activating of the stirt1/Nrf2 pathway and inhibiting NOD-like receptor thermal protein domain associated protein 3 (NLRP3) inflammasome-dependent inflammation (Mei et al., 2022; Shi et al., 2022a). In addition, the beneficial effects of flavones on hemorrhagic injuries were also investigated. In the study of Tan et al., autologous blood was injected into the brain of rats to create an intracerebral hemorrhage model. Then they administered luteolin or vehicle solution intraperitoneally to the rats. Compared with the control group, rats with luteolin treatment performed fewer neurobehavioral abnormalities after 24 h and less brain water content after 72 h. Further investigation confirmed that these effects were related to the activation of the p62/Keap1/Nrf2 pathway (Tan et al., 2019). In another rat model, luteolin inhibited subarachnoid hemorrhage (SAH)-induced oxidative damage and neuroinflammation, evidenced by suppressed proinflammatory cytokine release and restored endogenous antioxidant systems. Meanwhile, luteolin mitigated neuronal degeneration and NLRP3 upregulation in an *in vitro* SAH model. Surprisingly, these protective effects were abrogated by ML385, providing evidence that luteolin exerts cerebral-protective effects against SAH largely depending on Nrf2 activation (Zhang et al., 2021).

#### 4.1.3 Other neuroprotective effects

Fu et al. demonstrated that apigenin could reduce the cerebral infarction area and improve prognosis after hypoxic-ischemic encephalopathy (HIE) in neonatal rats. Besides, it inhibited cell apoptosis in rats’ brain tissue by activating PI3K/AKT/Nrf2 pathway (Fu et al., 2021). Apigenin could also delay the D-galactose-induced aging process, such as alleviating aging-related motor dysfunction and memory impairments. These effects depended on the activation of the Nrf2 pathway (Sang et al., 2017). Xu et al. indicated that luteolin pre-treatment improved the recovery of motor performance and reduced cerebral edema in mice after traumatic brain injury (TBI). However, luteolin failed to protect against brain injury in Nrf2-deficient mice. Except for protective effects *in vivo*, luteolin upregulated the expression of Nrf2, Ho-1, and NQO1, lowered the intracellular ROS level, and ameliorated apoptotic cell ratio in neurons after scratch stimulation (Xu et al., 2014). Interestingly, Wang et al. orally administered wogonin to rats before and after the rats were exposed to a whole-body single dose of γ-irradiation. There were significant increases in Malondialdehyde (MDA) and NF-κB expressions but decreases in GSH, SOD, CAT, Nrf2, and Ho-1 expressions in the irradiated group. Wogonin reversed these adverse changes and ameliorated gamma irradiation-induced histopathological changes in rats’ brains (Wang et al., 2020a). These results showed that flavones have extensive pharmaceutical value for treating and preventing nervous system diseases by alleviating OS and inhibiting apoptosis and inflammation.

### 4.2 Respiratory protective effects

Tsai and co-workers demonstrated that baicalein could alleviate LPS-induced Acute lung injury (ALI), reflected in the alleviation of histopathological damage, the decrease of lung wet/dry (W/D) ratios, and the prevention of inflammatory substances infiltration. These effects may stem from its anti-inflammatory and antioxidant properties mediated by blocking the NF-κB pathway and activating the Nrf2 signaling pathway (Tsai et al., 2014). Yang and co-workers found that chrysin pretreatment alleviated carrageenan-induced pleurisy and lung injury by attenuating OS, preventing the generation of pro-inflammatory cytokines (TNF-α, IL-1β) and vascular cell adhesion molecules (VCAM-1 and ICAM-1) and furtherly inhibiting polymorphonuclear neutrophils. These were achieved through the activation of Nrf2 nuclear accumulation promoter Sirt1 and the inhibition of NF-кBp65 phosphorylation (Yang et al., 2018a). Cigarette smoke (CS) is a source of potent oxidants and a significant cause of respiratory inflammatory diseases, such as chronic obstructive pulmonary disease (COPD). Li and colleagues demonstrated that oroxylin A significantly protects against CS-induced lung inflammation. The study proved that oroxylin A effectively attenuated CS-induced lung histopathologic changes and cytokine secretion, including TNF-α, IL-1β, and Monocyte chemoattractant protein-1 (MCP-1). The remarkable protective effect of oroxylin A is attributable to the potent suppression of CS-induced oxidative stress by activating the Nrf2 pathway. Moreover, oroxylin A demonstrated significant *in vitro* protection of epithelial cells and macrophages against CS-induced damage (Li et al., 2016a).

### 4.3 Hepatoprotective effects

#### 4.3.1 Protective effects against liver injury

Zhao et al. revealed that apigenin potentially reversed liver toxicity caused by Acetaminophen (APAP) in C57BL/6 mice and L-02 cells. They found that apigenin activated the Nrf2 pathway and suppressed the transcriptional activation of nuclear p65, which may partly contribute to the hepatoprotective effects (Zhao et al., 2020). He and co-workers evaluated the hepatoprotective effects of five flavonoids, including apigenin, luteolin, and chrysin. The treatment of these compounds consistently performed hepatoprotective effects against LPS/D-GalN-induced Acute liver failure (ALF), in which apigenin had the most potent protective capacity. The underlying mechanism is implicated in upregulating the expression of Nrf2 and related antioxidant proteins, scavenging free radicals, suppressing inflammatory factors in the NF-κB pathway, and inhibiting hepatocyte apoptosis (He et al., 2019). Besides, Zhou et al. showed that baicalein had protective effects against hepatic I/R injury in mice by regulating the Nrf2/ARE pathway, and Nrf2 inhibitor ML385 could reverse the hepatic protective effects (Zhou et al., 2021a).

#### 4.3.2 Protective effects of high-fat diet (HFD) related liver diseases

Lipid metabolic disorders and OS are characteristics of nonalcoholic fatty liver disease (NAFLD). NAFLD is the hepatic manifestation of metabolic syndrome (MS) and is associated with many chronic liver diseases (Feng et al., 2017). Xin et al. found that treatment with baicalein attenuated nonalcoholic steatohepatitis induced by a methionine-choline-deficient (MCD) diet in rats via suppressing OS and inflammation and regulating the expression of fatty acid metabolism genes (Xin et al., 2014). Recently, Ke and co-workers reported that tangeretin supplementation retarded NAFLD progression in HFD-fed mice. Tangeretin attenuated hepatic OS and steatosis, improved glucose tolerance, regulated serum lipid levels (ALT, AST, TC, TG, HDL-C), and decreased the levels of inflammatory factors (IL-1β, IL-6, and TNF-α). These beneficial effects were associated with increasing the activities of phase II detoxifying enzymes via activating the hepatic Nrf2 pathway (Ke et al., 2022). PPARγ, a ligand-dependent transcription factor, undertakes pivotal roles in lipid metabolism and OS. In a previous study, Feng et al. demonstrated that apigenin could inhibit obesity-induced metabolic syndrome by binding and activating PPAR (Feng et al., 2016). Their further investigation indicated that Nrf2 activation induced by apigenin significantly attenuated HFD-induced NAFLD progression, including suppressing lipid accumulation and OS. However, Nrf2 activation inhibited apigenin from activating PPARγ, suggesting a novel regulatory mode of apigenin for NAFLD (Feng et al., 2017). Another study examined the impact of apigenin on MS induced by a high-fructose diet in mice. Their findings clearly demonstrated that apigenin activated the Nrf2 pathway, resulting in significant protective effects. Specifically, it improved insulin resistance, alleviated histological and functional changes in the liver, and prevented the alteration of serum lipid profile. (Yang et al., 2018b).

### 4.4 Reno-protective effects

Dai et al. found that baicalein had nephroprotective effects against colistin, an antibiotic with Nephrotoxicity. Baicalein co-administration markedly attenuated colistin-induced oxidative and nitrative stress in the kidneys of C57BL/6 mice. It inhibited inflammatory cell generation, decreased renal IL-1𝛃 and TNF-𝛂 levels, and caused less renal cell apoptosis. Meanwhile, baicalein co-administration upregulated nrf2 expression and downregulated NF-κB expression (Dai et al., 2017). Wang and co-workers reported that diosmetin ameliorated sepsis-induced acute kidney injury (AKI) in rats. Besides, diosmetin reduced LPS-induced inflammatory factors production and renal cell apoptosis, depending on the enhancement of the TUG1/Nrf2/HO-1 pathway (Wang et al., 2020b). Interestingly, Li et al. recently showed that baicalein could attenuate renal functional and histopathological impairment of BALB/c mice with pristane-induced lupus nephritis (LN). It is suggested that the relief may be due to the inhibition of myeloid-derived suppressor cell (MDSC) expansion and the modulation of the Nrf2/HO-1 axis and inflammatory signaling levels (NLRP3, NF-κB) in MDSCs (Li et al., 2019a).

### 4.5 Cardiovascular protective effects

#### 4.5.1 Protective effects of cardiac injury

OS has been linked to the development of several cardiovascular diseases. Several studies have demonstrated that some flavones could protect myocardial tissue and cardiomyocytes from OS-induced injury through mediating Nrf2-related molecular mechanisms (Cui et al., 2015; Sun et al., 2012; Xingyue et al., 2021). In a recent study, chrysin was suggested to manage the expression of the Nrf2-related antioxidant system, which is involved in the suppression of ER stress-induced apoptosis, thus protecting cardiac H9c2 cells against H_2_O_2_-induced injury (Yuvaraj et al., 2022a). Qi and colleagues found that luteolin treatment attenuated the cardiotoxicity of cisplatin by modulating Keap1/Nrf2 signaling pathway (Qi et al., 2022). Sahu et al. showed that baicalein restored doxorubicin-induced down-expression of myocardial antioxidants and increased the levels of Nrf2 and Ho-1. The adverse effects of doxorubicin, including myocardial NF-κB activation, increased Bax/Bcl-2 ratio, higher expression of P53 and cleaved caspase-3, all reversed by baicalein (Sahu et al., 2016).

#### 4.5.2 Vasoprotective effects

The increasing formation of low-density lipoproteins (LDLs) led by hypercholesterolemia plays an essential role in the progression of atherosclerosis. The link between hypercholesterolemia and OS was also implicated as a potential molecular mechanism behind the development of atherosclerosis to coronary heart diseases (CHD). Yuvaraj et al. found that chrysin presented anti-hyperlipidemic properties and effectively prevented atherosclerosis progression in hypercholesterolemia rats. It seems chrysin reduced serum lipid profile (TC, TG, LDL, and VLDL), decreased cell adhesion molecules ICAM and VCAM expression, and restored the morphological changes in the aorta and left ventricular myocardium. In essence, chrysin recovered the expression of Nrf2 and its related genes but attenuated inflammation and apoptosis in the aorta and myocardium tissues (Yuvaraj et al., 2021; Yuvaraj et al., 2022b). Similarly, acacetin not only significantly relieved oxidized-LDL-induced ROS accumulation and apoptosis in human endothelial cells via the MsrA-Keap1/Nrf2 pathway but also halted atherogenesis in apolipoprotein E deficiency (apoE−/−) mice via accelerating lipid metabolism and decreasing plasma inflammatory cytokines (Wu et al., 2021).

As a potent vasodilator, NO plays a vital role in vascular tone regulation. NO deficiency has been addressed to endothelial dysfunction and increased total peripheral resistance, resulting in hypertension. ROS such as superoxide (O2•−) can decrease NO bioavailability by reacting with NO to produce peroxynitrite (ONOO−). Therefore, the therapeutic values of antioxidants for hypertension deserve to be explored (Torok, 2008). Potue and co-workers demonstrated that nobiletin, a unique flavonoid exclusively found in citrus peels, had anti-hypertension effects in L-NAME-induced hypertensive rats. As is shown in their study, nobiletin relieved the extent of hypertension and recovered the function of endothelium-dependent vasorelaxation in perfused mesenteric vascular beds and thoracic aorta. Moreover, nobiletin decreased the production of vascular O_2_^−^ and plasma MDA and restored plasma NOx level, Nrf2/Ho-1 protein expressions, and eNOS. Vascular remodeling evidence, such as vascular morphology changes and collagen deposition observed in L-NAME rats, was alleviated by nobiletin, which was associated with inhibiting MMPs expression (Potue et al., 2019). Analogously, Meephat et al. focused on the anti-hypertension capacity of diosmetin in L-NAME-induced hypertensive rats. They found that a high dose (40 mg/kg) of diosmetin has a similar effect on reductions of blood pressure, vascular dysfunction, inflammation, and OS with captopril, a widely used hypertension drug. The molecular mechanisms were relevant to recovering the Nrf2/Ho-1 axis and downregulating the p-JNK/p-NF-κB protein expression (Meephat et al., 2021).

#### 4.5.3 Myocardium protective effects

In an *in-vitro* study, acacetin conferred significant cardiomyocytes protection against hypoxia/reoxygenation insult by scavenging ROS, suppressing the release of pro-inflammatory cytokines, and exerting anti-apoptosis property, via AMPK-mediated activation of Nrf2 signaling pathway (Wu et al., 2018). Yang and colleagues investigated the therapeutic function of luteolin for cardiac I/R injury in hypercholesterolemic rats. They observed severer impairment of myocardial functions, higher levels of myocardial MDA and LDH leakage, and more MMP loss of cardiac myocytes in the hypercholesterolemic group. Luteolin alleviated these injuries by activating PI3K/Akt/GSK3β/Nrf2 pathway (Yang et al., 2018c). The study conducted by Rani et al. presents concrete evidence that chrysin substantially enhances hemodynamic and ventricular dysfunction while effectively reducing infarct size in rats that have suffered from IR-induced myocardial infarction (MI). The protective effects of chrysin are made possible through the co-action of PPAR-γ and Nrf2, which successfully counteract the increase of apoptosis markers and NF-κB activation in the myocardium (Rani and Arya, 2020). Interestingly, luteolin co-treatment significantly promoted the protective effects of dexamethasone against MI injury compared with dexamethasone single administration, with the manner of mediating Keap1/Nrf2/Ho-1 pathway (Wang et al., 2022b).

Recently, some flavones were elucidated to protect against pressure overload-induced cardiac hypertrophy. Guo and colleagues established a pressure overload model in C57BL/6 mice by aortic banding. They found that diosmetin administration reduced cardiac hypertrophy and dysfunction and alleviated myocardial OS by upregulating endogenous oxidants. *In vitro*, diosmetin prevented cardiomyocytes from phenylephrine-induced hypertrophy. They demonstrated that diosmetin activated the PI3K/AKT pathway, promoting p62 accumulation and its interaction with Keap1, thus enabling Nrf2 nuclear translocation (Guo et al., 2022). Similarly, in neonatal cardiomyocytes from rats, acacetin could antagonize Angiotensin II-induced increase of myocyte surface area and ROS production. Acacetin upregulated cellular expression Nrf2 and its downstream genes and regulated inflammation- and apoptosis-related genes IL-6, Bax, and Bcl-2. These benefit effects were also verified in SD rats with cardiac hypertrophy induced by abdominal aorta constriction. Further, the researchers showed that activation of Sirt1/AMPK may be essential in acacetin-induced nrf2 activation and cardiac protection (Cui et al., 2022).

### 4.6 Anti-diabetes mellitus and its related complications

As is known, hyperglycemia is a significant risk factor for the development of diabetes, which can impair the function of *ß*-cells, resulting in OS and cell death. *ß*-cell mass reduction subsequently decreases insulin production and contributes to the progression of diabetes. Vitexin (apigenin-8-C-glucoside), a flavone c-glycoside, could protect pancreatic *ß*-cells from high-glucose-induced apoptosis and improve the insulin release and sensitivity via activating the Nrf2/ARE pathway, suppressing NF-κB signaling pathway, and promoting phosphorylation of insulin receptors (Ganesan et al., 2020). Protein glycation in the body can lead to dysfunction of intracellular and extracellular proteins. Advanced glycation end products (AGEs) are a group of heterogeneous compounds generated from protein glycation, in which reactive carbonyl species (RCS) have been identified as key intermediates. AGEs accumulation in the human body may impair NO signaling and induce OS, which has been implicated in the development and progression of diabetes. Therefore, impacting AGEs formation from RCS and ameliorating AGEs-induced OS and inflammation are perspectives for preventing diabetic complications. A recent study provided evidence that apigenin could effectively scavenge methylglyoxal (MG) and react with it to form adducts, furtherly inhibiting the formation of AGEs in human umbilical vein endothelial cells (HUVECs). Interestingly, both apigenin and its adduct could significantly inhibit AGEs-induced ROS production and inflammatory response in HUVECs via upregulating of Nrf2 and its downstream antioxidant molecules and inhibiting P65 phosphorylation and the secretion of pro-inflammatory mediators (Zhou et al., 2019). This evidence indicated that flavones could essentially inhibit the development of diabetes.

In specific conditions, flavones have therapeutic potential for many diabetic complications. For instance, cytotoxicity of MG has been demonstrated to involve in diabetes-associated bone defects. Luteolin activated the Nrf2/Ho-1 axis, thus suppressing MG-induced osteoblastic MC3T3-E1 cell death and attenuating the generation of intercellular ROS, TNF-α, and mitochondrial superoxide. Therefore luteolin may help prevent the development of diabetic osteopathy (Suh et al., 2016). Besides, excessive ROS generation under Type 1 diabetes mellitus (T1DM) condition is adverse to the functional fulfillment of mesenchymal stromal cells (MSCs), which are essential in bone healing. Li et al. found that chrysin improved the osteogenic differentiation capacity of bone-derived MSCs (BMSCs) exposed to high glucose circumstances, and accelerated bone healing in the calvarial bone defect model of rats. These beneficial results were partly due to the activation of PI3K/AKT/Nrf2 pathway (Li and Wang, 2022). Periodontitis has been proven to be closely associated with DM, in which OS was considered an important pathogenic factor contributing to chronic periodontitis in DM patients (CPDM). Experimental CPDM models were established in human gingival epithelial cells (hGECs) and SD rats by stimulating high levels of glucose and lipopolysaccharide. Baicalein restored CPDM-induced ROS production antioxidant enzymes and p-Nrf2 downregulation in hGECs, Besides, baicalein mitigated the alveolar bone loss in CPDM rats by increasing the level of p-Nrf2 in the periodontal tissue (Zhu et al., 2020).

In other ways, Xiao et al. discovered that luteolin could attenuate I/R injury of the myocardium by restoring ventricular function after reperfusion in diabetic rats. They showed that luteolin upregulated the expression of endothelium Nitric Oxide Synthase (eNOS) and sestrin2, which promoted the removal of Keap1 and furtherly activated the Nrf2-regulated antioxidative signaling pathway (Xiao et al., 2019; Zhou et al., 2021b). Zhang et al. studied the protective impact of apigenin against diabetic nephropathy. They found that apigenin treatment can attenuate high glucose-induced OS, inflammation, and apoptosis in renal tubular epithelial cells by activating the PI3K/AKT/Nrf2 signaling pathway (Zhang et al., 2019). What is more interesting, Li et al. encapsulated apigenin into solid lipid nanoparticles (SLNPs) through the micro-emulsification method to improve the bioavailability of apigenin. They found apigenin-SLNPs reduced histopathological changes in the renal tissue of streptozocin-induced diabetic mice. It raised the activity of Nrf2, Ho-1 SOD, and CAT and decreased the expression of MDA and NF-κB (Li et al., 2020). Taken together, flavones are promising candidates to be developed into potential agents for preventing and treating diabetic complications.

### 4.7 Cancers

As both Nrf2 activators and Nrf2 inhibitors, flavones can exert anti-cancer effects. For instance, Son et al. demonstrated that luteolin activated Nrf2 to inhibit Cr (VI)-induced ROS production in normal BEAS-2B cells, a human lung bronchial epithelial cell line, consequently preventing Cr (VI)-induced malignant transformation in normal cells. By contrast, in Cr (VI)-transformed malignant cells with low ROS levels and resistance to apoptosis, luteolin administration decreased constitutively activated Nrf2 and its target anti-apoptotic and antioxidant proteins, including Bcl-2, Bcl-XL, and HO-1 (Son et al., 2017). Zuo et al. found that luteolin blocked cell growth and colony formation of human colorectal adenocarcinoma HCT116 cells via activating Nrf2, Ho-1, and NQO1; Paredes-Gonzalez et al. found apigenin exhibited cytotoxicity and restored the expression of Nrf2 in the preneoplastic epidermal JB6 P + cell line. According to their studies, the activation of the Nrf2 pathway was caused by the demethylation of CpG sites in the Nrf2 promoter region and the suppression of DNA methyl-transferases (DNMTs) and histone deacetylases (HDACs) expression (Zuo et al., 2018; Paredes-Gonzalez et al., 2014).

On the other hand, as Nrf2 inhibitors, flavones could induce apoptosis or render the tumor cells more sensitive to therapeutic drugs. Tsai and co-workers showed that luteolin inhibited the stemness capacity of breast cancer cells and enhanced the cytotoxicity of the chemotherapeutic drug Taxol through downregulating Nrf2 expression. Nobiletin inhibited migration and proliferation but promoted apoptosis of human breast cancer cells via Nrf2, p38 MAPK, and NF-ΚB pathway (Tsai et al., 2021; Liu J. et al., 2018). Chen et al. discovered that diosmetin selectively induced apoptosis and enhanced the chemotherapeutic efficacy of paclitaxel in non-small cell lung cancer cells by disrupting PI3K/Akt/GSK-3β/Nrf2 pathway and leading to the accumulation of ROS. Besides, diosmetin impaired the growth of tumor cells and increased the treatment efficacy of paclitaxel in bearing mice (Chen et al., 2019). Gao and his colleagues demonstrated that apigenin and chrysin co-administration effectively sensitized chemo-resistant hepatocellular carcinoma cells to doxorubicin by suppressing the PI3K/Akt/Nrf2 pathway. Furthermore, apigenin was found to significantly decrease Nrf2 expression in tumor tissue and enhance the sensitivity of tumor xenografts to doxorubicin (Gao et al., 2013a; Gao A. et al., 2013). Feng et al. found significantly lower GSK3β levels of skin tissue in patients with melanoma compared with normal tissue. Interestingly, nobiletin could increase the expression of GSK3β and further inhibit the Keap1/Nrf2/HO-1 signaling pathway in human melanoma cells. Therefore, the carcinoma cells were triggered to ferroptosis, increased lipid peroxidation and ROS production, GSH depletion, GPX4 (an indicator of ferroptosis) inactivation, and iron accumulation (Feng et al., 2022). Kim et al. demonstrated the significant impact of wogonin in sensitizing cisplatin-resistant human head and neck cancer (HNC) cells *in vivo* and *in vitro*. They discovered that the suppression of the Nrf2-GSTP1 axis by wogonin could activate JNK and PARP, leading to increased apoptosis of HNC cells (Kim et al., 2016). Besides, luteolin induced intracellular ROS accumulation in cholangiocarcinoma cells via inhibiting the Nrf2 pathway, resulting in mitochondrial-mediated cell death (Kittiratphatthana et al., 2016). Chrysin inhibited glioblastoma cell proliferation, migration, and invasion *in vivo* and *in vitro* in dose-dependent manners through the interference of the ERK/Nrf2 pathway (Wang et al., 2018). To sum up, flavones had chemo-preventive, antitumoral effects and effects of chemotherapeutic assistance for many types of cancers.

### 4.8 Optical protective effects

Age-related macular degeneration (AMD) is a primary reason for blindness in the elderly, and no effective treatment options exist until now, especially for dry AMD. Much evidence has shown that the primary etiology of AMD is oxidative damage of retinal pigment epithelium (RPE) (Zhang et al., 2020). Xu et al. previously found that treating human RPE cells with apigenin reduced t-BHP-induced oxidative injury and apoptosis by activating Nrf2 and its target genes. In contrast, the protection did not appear in cells with nif2 siRNA transfection (Xu et al., 2016). Zhang et al. produced the solid dispersion of apigenin, which had a higher solubility and bioavailability. They found it upregulated anti-oxidative enzyme activities and promoted autophagy in an Nrf2-dependent manner to suppress pathological changes of the retina in a dry AMD model of mice (Zhang et al., 2020). Recently, Chen and colleagues indicated that luteolin inhibited oxidative injury-induced epithelial-mesenchymal transformation in human RPE cells through AKT/GSK-3β pathway suppression, which relies on Nrf2 pathway activation. This provided a novel prospect for dry AMD treatment (Chen et al., 2022). Besides, Wang and co-workers clarified that nobiletin had therapeutic value for glaucoma. The results indicated that nobiletin significantly protected RGCs from hypoxia-induced apoptosis *in vitro*. Moreover, nobiletin effectively relieved OS in RGCs, restored RGC dysfunction, improved inner retinal structure, and mitigated Müller glial activation in a rat model of ocular hypertension. These effects were partly achieved through the activation of the Nrf2/Ho-1 pathway (Wang et al., 2022c).

### 4.9 Intestinal protective effects

A couple of studies by Yuan et al. reported that luteolin had repairing effects on the intestinal epithelial barrier dysfunction disrupted by toxicological substances, such as BDE-209 and ethanol. These protective effects may be related to regulating ERK/NF-κB/myosin light chain kinase (MLCK)-mediated expression of tight junction (TJ) proteins (ZO-1, occludin, and claudin-1) as well as Nrf2/ARE-regulated antioxidant pathways (Yuan et al., 2021a; Yuan et al., 2021b). Luteolin administration could also prevent dextran sulfate sodium (DSS)-induced colitis due to activation of the Nrf2 pathway, which subsequently enhances antioxidant capacity and inhibits proinflammatory mediators including iNOS, TNF-α, and IL-6 in the colon (Li et al., 2016b). Similarly, Li et al. demonstrated that diosmetin observably ameliorated histological damage of colon tissue in C57BL/6 mice with DSS-induced colitis. Diosmetin increased the expression of TJ proteins, inhibited the secretion of proinflammatory factors, and promoted the expression of GSH-Px, SOD, MDA, and GSH in colon tissue. *In vitro*, diosmetin alleviated LPS-induced intestinal epithelial barrier injury and OS in Caco-2 and IEC-6 cells. Their study further clarified that diosmetin activated the circ-Sirt1/Sirt1 axis, thus increasing Nrf2 expression and inhibiting NF-κB acetylation *in vivo* and vitro (Li et al., 2022b).

### 4.10 Osteoarticular protective effects

Evidence has shown that ROS can regulate the formation and function of osteoclasts, subsequently accelerating bone resorption and contributing to bone loss. Excessive bone erosion by osteoclasts is associated with many bone disorders. Xian and co-workers demonstrated that oroxylin A could suppress RANKL-induced osteoclastogenesis by inhibiting RANKL-induced ROS accumulation in an Nrf2-dependent manner and downregulating the activity of NFATc1, the master transcriptional regulator of RANKL-induced osteoclastogenesis. *In vivo*, oroxylin A performed anti-osteoclastogenic properties consistent with its *in vitro* effect, evidenced by it prevented post-ovariectomy (OVX)- and LPS-induced bone loss in C57BL/6 J mice (Xian et al., 2021).

Osteoarthritis (OA) is a chronic joint degenerative disease pathogenically featured by progressive chondrocyte apoptosis and extracellular matrix (ECM) degradation, inextricably interrelated inflammation and OS played significant roles in these processes (Zhou et al., 2022; Shi et al., 2022b). Recently, Zhou et al. showed that luteolin performed multiple protective effects on H_2_O_2_-treated chondrocytes, including inhibiting cell apoptosis, decreasing ECM degradation, and reducing the production of ROS and inflammatory mediators. Through Nrf2 and AMPK silencing and depletion, they proved these effects were attributed to the activation of AMPK and Nrf2 signaling. Moreover, in the destabilization of the medial meniscus (DMM) mouse model, luteolin treatment attenuated histological injury and downregulated MMP-13 level in cartilage (Zhou et al., 2022). Besides, Shi and co-workers found that tangeretin could slow down OA progression through attenuating inflammation and ECM degradation in IL-1β-treated chondrocytes and DMM mice, relying on mediating Nrf2/NF-κB and MAPK/NF-κB pathways (Shi et al., 2022b). Tangeretin also had therapeutic effects on rheumatoid arthritis (RA), it suppressed arthritis severity in rats with collagen-induced RA, decreased the production of MDA and pro-inflammatory factors but enhanced the expression of IL-10 and Nrf2-related antioxidant enzymes (Li et al., 2019b). Wang et al. established intervertebral disc degeneration (IDD) models by treating nucleus pulposus cells (NPCs) with TBHP *in vitro* and puncturing coccygeal intervertebral discs *in vivo*. They found acacetin had beneficial effects for IDD treatment both *in vivo* and *in vitro*. The mechanisms may be related to Nrf2 signaling activation and inhibition of the MAPK pathways (Wang et al., 2020c). Analogously, wogonin relieved IL-1β induced inflammation in NPCs via activating the Nrf2/ARE pathway and inhibiting MAPK pathways. Intravertebral injection of wogonin also improved histological structure and mitigated the progression of IDD in the rat model (Fang et al., 2018).

### 4.11 Reproductive protective effects

Jiang et al. established an endometritis model via giving LPS (2.5 mg/mL) to the mouse uterus, apigenin was given intraperitoneally 1 h before LPS treatment and 24 h later. They noted that apigenin activated Nrf2/Ho-1 axis in the uterus tissues and relieved LPS-induced NF-κB activation, inflammatory cytokine production, and MDA accumulation, thus relieving endometritis. (Jiang et al., 2018). Huang et al. discovered that luteolin can improve rats’ polycystic ovary syndrome (PCOS) symptoms by reducing insulin resistance and enhancing antioxidative response. This is achieved by triggering the PI3K/AKT and Nrf2 signaling pathways (Huang and Zhang, 2021). Ma and colleagues found that in models of triptolide-induced testis injury, luteolin prevented the impairment of BTB permeability by upregulating the expression of connexin43 (Cx43). Besides, it inhibited ROS accumulation and cell apoptosis by activating the Nrf2 pathway, thereby ameliorating testicular damage and spermatogenesis disturbance (Ma et al., 2019). Supplementary Table S1 summarized Nrf2-related therapeutic effects of flavones.

### 4.12 Protective effects against environmental toxicants

Humans are continuously exposed to environmental toxicants, especially individuals under occupational exposure (Mahdiani et al., 2022). Until now, flavones have been reported to protect multiple organs against many toxicants. *In vivo*, apigenin treatment mitigated SiO2-caused lung histopathological injury by promoting the Nrf2 pathway and decreasing the ratio of Bax/Bcl-2. *In vitro*, apigenin incubation prevented cellular ROS accumulation and rescued A549 cells from SiO2-induced apoptosis (Wang et al., 2022d). Liu et al. found that luteolin was a potent candidate for HgCl_2_-elicited lung injury by activating AKT/Nrf2 pathway and inhibiting NF-κB activation. Their results showed that luteolin not only attenuated HgCl_2_-induced pulmonary histologic conditions but also suppressed neutrophil recruitment in the lung tissue of mouse (Liu et al., 2018b).

The liver is believed to be another target organ for various toxicants. Yang et al. reported that luteolin could alleviate the histopathological damage in liver tissue induced by HgCl_2_. They revealed that luteolin inhibited inflammation and apoptosis in hepatocytes by activating SIRT1/Nrf2 pathway, and inhibiting NF-κB and P53 (Yang et al., 2016). Besides, luteolin single administration effectively diminished CCl_4_-caused hepatotoxicity by activating Nrf2 and Ho-1, suppressing inflammatory cytokines expression and apoptosis. Furtherly, co-administration with metformin significantly enhanced these effects, suggesting luteolin may have a synergistic defensive impact with other drugs (Yan et al., 2019). Besides, Rajput and co-workers demonstrated that luteolin delivery significantly ameliorated Aflatoxin B1 (AFB1)- elicited growth retardation and liver tissue injury in mice via Nrf2-mediated ROS scavenging and mitochondrial apoptosis inhibition (Rajput et al., 2021). Recently, Deng and co-workers explored the mechanisms of liver fibrosis in quails after long-term exposure to a new nicotine insecticide, Imidacloprid (IMI). Their results revealed that IMI can induce liver fibrosis. It observably changed the fibrosis-related genes in quail livers, leading to hematologic toxicity, hepatic steatosis, inflammation, and downregulation of the Nrf2 pathway. However, as an Nrf2 activator, luteolin markedly reversed these changes (Deng et al., 2022).

Several flavones such as luteolin, could also alleviate nephrotoxicity induced by several toxicants. Pretreating these flavones recovered serum levels of renal injury biomarkers such as urea and creatinine and attenuated histopathological changes of kidneys after acute injury. The mechanisms included 1) activating the Nrf2/ARE pathway, and amending the decline of antioxidant gene expressions, such as Ho-1 and NQO1 2) suppressing pro-apoptosis molecule P53, and regulating apoptotic related Bax, caspase-3 and Bcl-2. 3) suppressing NF-κB pathway and downregulating the expression of inflammatory cytokines (Tan et al., 2018; Albarakati et al., 2020).

Lead (Pb) is a prevalent pollutant that harms the male reproductive system. It causes testicular injury by promoting the accumulation of lipid peroxidation (LPO) and NO, depleting the antioxidant capacity (GSH, CAT, GR, GPx), increasing the mRNA levels of inflammatory markers such as TNF-α, IL-1β, and Nos2, and increasing apoptosis activities in rats. Surprisingly, luteolin pre-treatment upregulated the expression of Nrf2 and Ho-1, thus alleviating the toxicity of Pb to protect against pb-induced testis injury and blood–testis barrier (BTB) disruption (Al-Megrin et al., 2020). In addition, Yu and colleagues demonstrated that wogonin can help to improve the production of steroids and sperm in rats with cadmium-caused testicular injury. This is achieved by regulating oxidative status, inflammation, and apoptotic mediators (Yu et al., 2020). Supplementary Table S2 summarizes these protective functions against environmental toxicants.

## 5 Discussion

OS, inflammation, and apoptosis are essential factors in the pathogenesis of diseases such as neurodegenerative diseases, diabetes, cancers et al., and an intimate correlation exists among the three processes. Nrf2 regulation is necessary to maintain cellular homeostasis against OS and inhibit the development of many pathological conditions. Throughout this review, we learned that Nrf2 can be pivotal in inflammation regulation and summarized the interaction between Nrf2 and NF-κB. Additionally, Nrf2 activation could also influence the process of apoptosis, as ROS stimulation directly or indirectly participates in the three apoptotic pathways. With these concepts, exploring agents which can target OS via regulating Nrf2 can be perspective in clinical.

In recent years, phytochemicals with lower side effects have attracted wide attention. Flavones, an essential subclass of the flavonoid family present in fruits and vegetables, can potently regulate Nrf2/ARE pathway to exhibit their protective properties, including anti-oxidation, anti-inflammation, and anti-apoptosis. Therefore, they can be compelling therapeutic agents for various OS-related disorders. However, there is a limited number of human studies in this field, and Nrf2-related protective effects of flavones still need to be investigated and confirmed in human subjects. On the other hand, these plant-derived chemicals may also need to improve their solubility and bioavailability for clinical application. Nanocarriers such as liposomes, nano-emulsions, micelles, phytosomes, and nanoparticles have been synthesized to enhance the therapeutic efficiency of flavones (Davaran et al., 2018; Ju et al., 2022; Telange et al., 2017; Ganguly et al., 2021; Huang et al., 2017). For example, Ju et al. used docosahexaenoic acid-enriched phosphatidylcholine as an emulsifier to establish a delivery system for nobiletin. The prepared nobiletin-loaded nano-emulsion had 3.5 times more bioavailability than nobiletin oil suspension *in vitro*. Besides, the emulsion was more easily absorbed *in vivo* and presented significantly higher concentration in the liver, brain, kidney, spleen, and heart (Ju et al., 2022). Ganguly S and colleagues fabricated apigenin-galactosylated-PLGA-nanoparticles, which had significantly high cytotoxic and apoptotic potentials for human hepatocellular carcinoma, compared with original apigenin (Ganguly et al., 2021). Therefore, developing a novel delivery system of flavones will pave the road for their application in the clinical area.

## 6 Conclusion

In this review, we learned that flavones can be important therapeutic agents for various OS-related disorders. Nrf2/ARE is a compelling target for these phytochemicals to exhibit their protective properties, including anti-oxidation, anti-inflammation, and anti-apoptosis. These flavones in aglycone form could regulate Nrf2 expression by several mechanisms: 1) dissociating the binding between Nrf2 and Keap1 via PKC-mediated Nrf2 phosphorylation and P62-mediated Keap1 autophagic degradation; 2) regulating Nrf2 nuclear translocation by various kinases like AMPK, MAPKs, Fyn; 3) decreasing Nrf2 ubiquitination and degradation via activating sirt1 and PI3K/AKT-mediated GSK3 inhibition; and 4) epigenetic alternation of Nrf2 such as demethylation at the promoter region and histone acetylation. However, further investigations are needed to enhance the biological activities of this kind of phytochemicals.
