# Supplementary material for: Nrf2-mediated therapeutic effects of dietary flavones in different diseases
Source: Front Pharmacol. 2023 Sep 12;14:1240433. doi: 10.3389/fphar.2023.1240433 (PMC10520786; doi:10.3389/fphar.2023.1240433)
Supplement: Supplementary file 1 [file Table1.docx]

Supplementary Material

Nrf2-mediated therapeutic effects of dietary flavones in different diseases

**Wenkai Huang ^1^, Yuan Zhong ^1^, Botao Gao ^1^, Bowen Zheng ^2^, Yi Liu ^2,^** *****

**^1^** School and Hospital of Stomatology, China Medical University, Liaoning Provincial Key Laboratory of Oral Disease, Shenyang 110002, China

**^2^** Department of Orthodontics, School and Hospital of Stomatology, China Medical University, Liaoning Provincial Key Laboratory of Oral Disease, Shenyang 110002, China

*** Correspondence:**Yi Liu, Department of Orthodontics, School, and Hospital of Stomatology, China Medical University, Shenyang, 110002, China
[liuyi@cmu.edu.cn](mailto:liuyi@cmu.edu.cn)

# Supplementary Figures and Table

| Type of diseases | Type of flavones | Animal model | Cell line | dose | mechanisms | reference | |
| --- | --- | --- | --- | --- | --- | --- | --- |
| PD | Apigenin | Male Wistar rats | -- | 25, 50mg/kg | ↑p-nrf2, SOD, CAT, GSH ↓MDA;  ↓p-NF-κB, TNF-α, IL-1β, IL-6, and  ↓caspase-1 | [Patel & Singh, 2022] | |
| neurodegenerative diseases | Apigenin | -- | Murine BV2 microglia | 10, 20, 40μM | ↑p-GSK3𝛃, Nrf2, and HO-1;  ↓nuclear P65, TNF-α, IL-1ß, and IL-6 | [Chen *et al.*, 2020] | |
| PD | luteolin | -- | Murine BV2 microglia | 1-20μM | ↑nrf2; ↓IL-1𝛃 | [Elmazoglu *et al.*, 2020] | |
| PD | baicalein | -- | PC12 | 50, 100, 200μM | ↑P-PKCα, P-AKT; ↓Keap1 ↑nuclear nrf2, Ho-1, SOD, CAT ↓MDA; | [Zhang *et al.*, 2012] | |
| PD | chrysin | zebrafish and mice | PC12 | 3, 6, 12μM | ↑nuclear nrf2, Ho-1, SOD, CAT ↓ROS, MDA; ↓P-P65 ↓iNOS and NO | [Zhang *et al.*, 2015] | |
| AD | apigenin | -- | SH-SY5Y-derived cells | 5, 10μM | ↑nrf2↓ROS  ↓Bax ↑Bcl-2 | [Chiang *et al.*, 2021] | |
| AD | Nobiletin | male Wistar rats | -- | 10mg/kg | ↑nrf2, GSH, CAT, SOD ↓ROS, MDA, 3-nitrotyrosine (3-NT); ↓NF-ΚB, TLR4, TNF-α | [Ghasemi-Tarie *et al.*, 2022] | |
| Cerebral I/R  Injury | Apigenin | -- | PC12 | 1, 10, 20μM | ↑nrf2, HO-1, NQO1, SOD, CAT, GSH-Px ↓ROS; ↓P53 and its downstream genes  ↓mitochondrial apoptosis | [Guo *et al.*, 2014] | |
| Cerebral I/R  Injury | Baicalein | Male Wistar rats | SH-SY5Y | 2.5, 5, 10mg/kg;  4, 8μM | ↑nrf2 pathway; ↓NF-κB and other inflammatory mediators; ↓caspase-3 and Bax ↑Bcl-2; | [Yuan *et al.*, 2020] | |
| Cerebral I/R injury | diosmetin | Male SD rats | PC12 | 40-100mg/kg;  20-80nM; | ↑SIRT1 ↓Keap1 ↑nuclear nrf2, Ho-1, NQO1, SOD, CAT, GSH ↓MDA, ROS;  ↓cleaved Caspase 3 and cleaved PARP1; ↓NLRP3, IL-1β and IL-18 | [Mei *et al.*, 2022; Shi *et al.*, 2022a] | |
| Intracerebral hemorrhage (ICH) | luteolin | Male SD rats | rat cortical neurons | 10, 20mg;  10μM | ↓Keap1, P62; ↑nrf2 nuclear translocation, NQO1, Ho-1 | [Tan *et al.*, 2019] | |
| SAH | luteolin | Male SD rats | Primary cortical neurons and microglia | 60mg/kg;  25μM | ↑nuclear nrf2, Ho-1, SOD, GSH, GSH-Px ↓H2O2, MDA, 8-OHdG;  ↓NLRP3 inflammasome signaling | [Zhang *et al.*, 2021] | |
| HIE | Apigenin | Adult SD rats | -- | 20mg/kg | ↑PI3K/AKT pathway ↑nrf2, Ho-1;  ↓Bax, cleaved caspase-3 ↑Mcl-1, Bcl-2; | [Fu *et al.*, 2021] | |
| Aging | apigenin | male Kunming mice | -- | 50mg/kg | ↑nrf2 translocation, SOD, CAT, Ho-1 and NQO1 ↓MDA | [Sang *et al.*, 2017] | |
| TBI | Luteolin | Male ICR mice | Mice cortical neuronal cells | 30mg/kg;  10, 25μM | ↑nuclear nrf2, Ho-1, NQO1, GPx  ↓MDA, ROS | [Xu *et al.*, 2014] | |
| irradiation-induced brain injury | wogonin | male Wistar albino rats | -- | 30mg/kg | ↓NF-κB, TNF-α, IL-1β, IL-6;  ↑nrf2, Ho-1, GSH, SOD, CAT, GPx ↓MDA | [Wang *et al.*, 2020a] | |
| lung injury | Baicalein | Male SD rats | -- | 20mg/kg | ↑nuclear nrf2, Ho-1 ↓O_2_^_^, iNOS;  ↓nuclear P65 ↓MPO, TNF-α, IL-1β, IL-6 | [Tsai *et al.*, 2014] | |
| pleurisy and Lung injury | chrysin | Male SD rats | -- | 40mg/kg | ↑SIRT1, nuclear nrf2, Ho-1, GSH ↓MDA;  ↓P-P65, TNF-α and IL-1β ↓MPO activity ↓ICAM-1 and VCAM-1 | [Yang *et al.*, 2018a] | |
| COPD | Oroxylin A | Male C57BL/6 mice | BEAS-2B; RAW264.7 | 15-60mg/kg; | ↑nuclear nrf2, Ho-1, GPX-2, GR and GSH ↓OS markers; ↓TNF-α, IL-1β, and MCP-1 | [Li *et al.*, 2016a] | |
| Acute liver injury (ALI) | Apigenin | C57BL/6 mice | L-02 | 80mg/kg;  50μM | ↑SIRT1-mediated autophagy ↑nrf2 pathway ↓MDA, ROS; ↓nuclear P65 | [Zhao *et al.*, 2020a] |  |
| ALI | Apigenin;  luteolin;  Chrysin | Male Kunming mice | -- | 100mg/kg | ↑nrf2, Ho-1, CAT, SOD, and T-AOC ↓MDA; ↓nuclear P65, COX-2, IL-1β, iNOS;  ↑Bcl-2, Bax, cleaved Caspases 3,8 and 9 | [He *et al.*, 2019] |  |
| NAFLD | Baicalein | Male SD  rats | -- | 10mg/kg | ↑nrf2, Ho-1, CAT ↓MDA, ROS;  ↓NF-κB p65, TNF-α, IL-6, iNOS;  regulating fatty acid metabolism genes | [Xin *et al.*, 2014] |  |
| NAFID | Tangeretin | Male C57BL/6J mice | -- | 0.5% of diet | ↑nuclear nrf2, Ho-1, NQO1, GCLC, SOD, GSH ↓MDA | [Ke *et al.*, 2022] |  |
| MS | apigenin | male KM mice | -- | 50mg/kg | ↑nrf2 nuclear translocation, Ho-1, NQO1, SOD, CAT, and GPH-Px ↓MDA | [Yang *et al.*, 2018b] |  |
| Hepatic I/R Injury | Baicalein | male C57BL/6 mice | -- | 50, 100mg/kg | ↑nuclear nrf2, Ho-1, NQO1, SOD ↓ROS, MDA | [Zhou *et al.*, 2021a] |  |
| renal injury | Baicalein | C57BL/6 mice | -- | 50, 100mg/kg | ↑nrf2, Ho-1, SOD, CAT and GSH ↓MDA, iNOS and NO; ↓NF-κB, IL-1𝛃 and TNF-α; ↓caspase-9 and -3 | [Dai *et al.*, 2017] |  |
| AKI | Diosmetin | SD rats | CRL-2573 | 20μM | ↑lncRNA-TUG1 ↑nuclear nrf2, Ho-1, NQO1, SOD ↓MDA; ↓TNF-α, IL-1β, and IL-6; ↓caspase-3 | [Wang *et al.*, 2020b] |  |
| LN | Baicalein | Female BALB/c mice | MDSCs | 100mg/kg; 0.04μM | ↑nuclear nrf2, Ho-1, GPx ↓ROS, MitoSOX;  ↓NLRP3 and p-P65 ↓IL-1β and IL-18 | [Li *et al.*, 2019a] |  |
| Cardiac injury | chrysin | -- | H9c2 | 50μM | ↑nrf2, SOD, CAT ↓ROS; ↓ER stress signaling genes ↑MMP ↓Caspase-3 | [Yuvaraj *et al.*, 2022a] |  |
| Cardiac injury | luteolin | Male C57BL/6 mice | HL-1 | 20mg/kg;  1, 3, 10μM | ↓Keap1 ↑nrf2, Ho-1, NQO1, SOD ↓ROS, MDA; ↓Bax | [Qi *et al.*, 2022] |  |
| Cardiac injury | Baicalein | Male BALB/c mice | -- | 25, 50mg/kg | ↑nuclear nrf2, Ho-1, NQO1, GST, SOD, CAT, GSH ↓iNOS; ↑IκBα ↓nuclear P65; ↓Bax/Bcl-2, cleaved caspase-3 and PARP | [Sahu *et al.*, 2016] |  |
| atherosclerosis | chrysin | Male SD rats | -- | 100mg/kg | ↑AKT, nrf2, SOD, CAT, GCL3, Cav1, eNOS  ↓TBARS; ↓caspase-3 | [Yuvaraj *et al.*, 2021] |  |
| atherosclerosis | chrysin | Male Wistar rats | -- | 100mg/kg | ↑nrf2, SOD, CAT ↓ROS; ↓NLRP3, TLR4; ↓caspase-3; ↓ICAM and VCAM; | [Yuvaraj *et al.*, 2022b] |  |
| atherosclerosis | acacetin | Female ApoE−/− mice | EA.hy926 | 15mg/kg; 3umol/L | ↓Keap1 ↑MsrA, p-nrf2, Ho-1 ↓ROS, MDA; ↑IL-10 ↓IL-6, TNF-𝛼; ↓caspase-3, Bax ↑Bcl-2 | [Wu *et al.*, 2021] |  |
| hypertension | Nobiletin | Male SD rats | -- | 40mg/kg | ↑nrf2, Ho-1 ↓O2•-, MDA ↑NOx, eNOS; ↓MMP-2, MMP-9 | [Potue *et al.*, 2019] |  |
| hypertension | diosmetin | Male SD rats | -- | 20, 40mg/kg | ↑nrf2, Ho-1, SOD, CAT ↑NOx ↓O2•-, MDA;  ↓p-JNK, p-NF-κB, IL-6 | [Meephat *et al.*, 2021] |  |
| Cardiac injury | Acacetin | Male SD rats | neonatal rat cardiomyocytes; H9C2 | 10mg/kg; 1,3μM | ↑p-AMPKα ↑nrf2, Ho-1, SOD1, SOD2 ↓ROS; ↓IL-6 and TLR-4 ↑IL-10;  ↑Bcl-2 ↓Bax, cleaved caspase-3 | [Wu *et al.*, 2018b] |  |
| Cardiac I/R Injury | Luteolin | Male SD rats | -- | 100mg/kg | ↑p-Akt, p-GSK3β ↓nuclear Fyn  ↑nuclear nrf2, Ho-1, NQO1↓MDA; | [Yang *et al.*, 2018c] |  |
| Cardiac I/R Injury | Chrysin | Male Wistar rats | -- | 60mg/kg | ↑PPAR-γ, Nrf2, GSH and CAT ↓TBARs, 8-OHdG  ↓IKK-β, NF-κB, TNF-α, MPO and CRP | [Rani & Arya, 2020] |  |
| Cardiac hypertrophy | Diosmetin | male C57BL/6 mice | neonatal rat ventricular myocytes | 40mg/kg;  10, 50μM | ↑PI3K/AKT pathway, ↑p62;  ↑nuclear nrf2, Ho-1, NQO1, SOD2, GSH/GSSH | [Guo *et al.*, 2022] |  |
| Cardiac hypertrophy | acacetin | SD rats | Neonatal cardiomyocytes of rats | 10mg/kg;  1, 3μM | ↑Sirt1, p-AMPK ↑nuclear nrf2, Ho-1, SOD1, SOD2 ↓ROS;  ↑Bcl-2, PGC-1α and PPARα ↓Bax | [Cui *et al.*, 2022] |  |
| diabetic osteopathy | luteolin | -- | MC3T3-E1 | 0.1, 1μM | ↑nuclear nrf2, Ho-1, GSH ↓ROS; ↓TNF-α; ↓RAGE; improving mitochondrial dysfunctions | [Suh *et al.*, 2016] |  |
| Diabetic bone defect | chrysin | Male SD  rats | BMSCs | --;  1, 5μM | ↑PI3K/AKT pathway ↑nrf2, Ho-1, SOD ↓ROS, MDA | [Li & Wang, 2022] |  |
| CPDM | Baicalein | SD rats | hGECs | 50-200mg/kg; 0.01-0.5μg/ml; | ↑p-nrf2, CAT, GCLC, SOD1, SOD2 ↓ROS | [Zhu *et al.*, 2020] |  |
| Cardiac I/R injury of diabetic | luteolin | Male SD rats | -- | 100mg/kg | ↑eNOS and sestrin2 ↑nuclear nrf2, Ho-1, SOD, CAT, GPx ↓MDA, 8-OHdG; ↑MMP | [Xiao *et al.*, 2019; Zhou *et al.*, 2021b] |  |
| DN | apigenin | -- | HK-2 | 100, 200μM | ↑nrf2, Ho-1, SOD, CAT ↓MDA;  ↓TNF𝛼, IL-1𝛃, and IL-6; | [Zhang *et al.*, 2019] |  |
| DN | Apigenin SLNPs | Male rats | -- | 25, 50mg/kg | ↑nrf2, Ho-1, SOD, CAT ↓MDA;  ↓NF-κB, TNF𝛼, IL-1𝛃, IL-6 | [Li *et al.*, 2020] |  |
| Colorectal cancer | luteolin | -- | HCT116 | 15, 30μM | ↑nrf2, Ho-1, NQO1;  ↓DNMTs, HDACs | [Zuo *et al.*, 2018] |  |
| Skin cancer | apigenin | -- | JB6 P + | 1.56, 6.25μM | ↑nuclear nrf2, NQO1;  ↓DNMTs, HDACs | [Paredes-Gonzalez *et al.*, 2014] |  |
| Breast cancer | luteolin | -- | MDA-MB-231 | 1, 2μM | ↓nrf2, Ho-1, sirt3 | [Tsai *et al.*, 2021] |  |
| Breast cancer | nobiletin | -- | MCF-7 | 100, 200μM | ↑p-p38 ↓nuclear nrf2; ↓nuclear P65, MMP-9, MMP-2; ↓Bcl-2 ↑Bax, P53, caspase-3 | [Liu *et al.*, 2018b] |  |
| Lung cancer | diosmetin | Female BALB/c nude mice | A549, H1299 | 50mg/kg;  5, 10, 20μM | ↓p-AKT, p-GSK‐3β ↓nrf2, Ho-1, NQO1 ↑ROS; ↑Bax ↓Bcl-2 | [Chen *et al.*, 2019] |  |
| Hepatocellular carcinoma | apigenin | Male BALB/c nude mice | BEL-7402 | 50mg/kg;  10, 20μM | ↓PI3K/AKT pathway  ↓nrf2 signaling pathway | [Gao *et al.*, 2013a; Gao *et al.*, 2013b] |  |
| Cholangiocarcinoma | Luteolin | -- | KKU-100 | 25μM | ↓nrf2, Ho-1, 𝝲-GCL, GSH ↑ROS;  ↓MMP ↑cytochrome c release, caspase-9, caspase-3 ↓Bcl-2, Bcl-XL | [Kittiratphatthana *et al.*, 2016] |  |
| glioblastoma | chrysin | male BALB/c athymic nude mice | T98, U251, and U87 | 40,80mg/kg;  10, 30, 60μM | ↓p-ERK, nuclear nrf2, Ho-1, NQO1 | [Wang *et al.*, 2018] |  |
| Melanoma | nobiletin | -- | SK-MEL-28 | 15, 45μM | ↑GSK3β, Keap1 ↓nrf2, Ho-1, GSH ↑ROS, MDA | [Feng *et al.*, 2022b] |  |
| HNC | wogonin | male BALB/c athymic nude mice | Human HNC cell lines | 50 mg/kg; 100μM | ↓nrf2, NQO1, GSTP1 ↓GSH, GSSG ↑ROS; ↑apoptosis: ↑P-JNK, cleaved PARP, PUMA, Bax ↓Mcl-1; | [Kim *et al.*, 2016] |  |
| Dry AMD | apigenin | C57BL/6 mice | -- | 60mg/kg | ↑nuclear nrf2, NQO1, Ho-1, SOD, GSH-Px  ↓ROS, MDA; ↑autophagy: ↑P62, LC3 II | [Zhang *et al.*, 2020a] |  |
| Dry AMD | apigenin | -- | ARPE-19 | 400μM | ↑nrf2 nuclear translocation, Ho-1, NQO1, GCLM, SOD, CAT, GSH-Px, T-AOC ↓ROS, MDA | [Xu *et al.*, 2016] |  |
| Dry AMD | luteolin | -- | ARPE-19 | 20, 40mg/kg | ↑nuclear nrf2, NQO1, Ho-1, SOD, GSH-Px  ↓ROS, MDA; ↓p-AKT, p-GSK-3β; ↓EMT | [Chen *et al.*, 2022] |  |
| glaucoma | Nobiletin | male Wistar rats | RGCs of rats | 10μM | ↑nuclear nrf2, Ho-1 ↓8-OHdG | [Wang *et al.*, 2022d] |  |
| intestinal epithelial barrier damage | Luteolin | -- | Caco-2 | 5, 50, 100μM | ↑nuclear nrf2, NQO1, SOD, GSH ↓ROS; ↓TNF-α, IL- 6, and IL-1β; ↓ERK/NF-κB/MLCK pathway; ↑TJ proteins | [Yuan *et al.*, 2021a; Yuan *et al.*, 2021b] |  |
| UC | Luteolin | Male  C57BL/6 mice | -- | 20, 50mg/kg | ↑nuclear nrf2, Ho-1, NQO1, SOD and CAT  ↓MDA; ↓iNOS, TNF-α and IL-6 | [Li *et al.*, 2016b] |  |
| UC | Diosmetin | C57BL/6 mice | Caco-2 and IEC-6 | 25, 50mg/kg; 25-100μM | ↑circSirt1, Sirt1; ↑nuclear nrf2, Ho-1, SOD, GSH, GSH-Px ↓MDA, ROS; ↓acetylated NF-ΚB, IL-1β, IL-6, COX-2, and IFN-γ | [Li *et al.*, 2022] |  |
| osteolytic diseases | Oroxylin A | C57BL/6 mice | BMMs of mice | 5mg/kg;  5, 10μM | ↑nuclear nrf2, Ho-1, CAT, GCLC ↓ROS; ↓osteoclast-related genes: NFATc1, c-Fos, CTSK, and ATP6V0D2 | [Xian *et al.*, 2021] |  |
| OA | Luteolin | Male C57BL/6 mice | murine chondrocytes | 10mg/kg;  10, 20μM | ↑AMPK/ nrf2 pathway, ↑GSH/GSSG ↓ROS;  ↓Cyto-C release ↓caspase-3;  ↓inflammatory mediators | [Zhou *et al.*, 2022] |  |
| OA | Tangeretin | Male C57BL/6 mice | mice chondrocytes | 10, 20mg/kg;  10, 20μM | ↑nrf2 nuclear translocation, Ho-1 ↓ROS; ↓MAPK pathways ↓nuclear p65, TNF-α, IL-6, NO, PGE2; ↓ECM degradation: ↓MMP-13, ADAMTS5 ↑collagen II; | [Shi *et al.*, 2022b] |  |
| RA | Tangeretin | Male Wistar rats | -- | 50mg/kg | ↑nrf2, NQO1, SOD, CAT, GSH ↓MDA; ↓IL-1β, TNF-α, IFN-γ, PGE2, MPO, NO ↑IL-10; | [Li *et al.*, 2019c] |  |
| IDD | Acacetin | Male  SD rats | Primary NPCs | 25mg/kg;  0.3, 1μM | ↑nuclear nrf2, Ho-1, NQO1, SOD ↓ROS;  ↓p-p38, p-ERK, p-JNK; ↓iNOS, COX-2; ↑collagen II ↓MMP13, MMP9 and MMP3; | [Wang *et al.*, 2020c] |  |
| IDD | Wogonin | Male  SD rats | rat NPCs | 50uM;  10-50μM | ↑nrf2, SOD-2, GCLC, NQO-1 and HO-1;  ↓P-P38 and P-JNK ↓IL-6, COX-2 and iNO; ↓MMP-1, MMP-3, MMP-13, and ADAMTS-4; | [Fang *et al.*, 2018] |  |
| Endometritis | apigenin | Female C57BL/6 mice | -- | 10, 20, 40mg/kg | ↑nrf2, Ho-1 ↓MPO, MDA;  ↓p-P65 and p-IκBα, TNF-α and IL-1β; | [Jiang *et al.*, 2018] |  |
| PCOS | luteolin | Female SD rats | -- | 50mg/kg | ↑PI3K/AKT pathway; ↑nrf2, HO-1, NQO1, GSH, SOD, CAT, and GSH-Px; | [Huang & Zhang, 2021] |  |
| Testis Injury | Luteolin | male ICR mice | Primary SCs and  TM4 | 20, 40mg/kg;  5, 10μM | ↑nuclear nrf2, NQO1, Ho-1, GSH ↓ROS, MDA;  ↓mitochondrial-mediated apoptosis; ↑Cx43 | [Ma *et al.*, 2019] |  |

# Supplementary Table 1: Nrf2-related therapeutic effects of flavones

| toxicants | flavones | Animal model | Cell line | dose | mechanisms | reference | |
| --- | --- | --- | --- | --- | --- | --- | --- |
| SiO2-induced Lung injury | Apigenin | C57BL/6 mice | A549 | 20mg/kg;  25μM | ↑nrf2, Ho-1, γ-GCSc and SOD ↓ROS, MDA; ↓Bax, cleaved caspase 3 ↑Bcl-2; | [Wang *et al.*, 2022b] | |
| HgCl_2_-induced Lung injury | Luteolin | male Kunming mice | -- | 100mg/kg | ↑p-AKT, nuclear nrf2, Ho-1, NQO1, SOD, GSH ↓MDA; ↓nuclear NF-κB, TNF-α, IL-6, IL-1β ↓MPO activity; ↓Bax, caspase-3 ↑Bcl-2 | [Liu *et al.*, 2018a] | |
| HgCl_2_-induced Liver injury | Luteolin | male Kunming mice | Hepatocytes of mice | 100mg/kg;  20μM | ↑Sirt1, nrf2, Ho-1, NQO1, GSH ↓MDA, ROS  ↓NF-κB, TNF-α; ↓P53, Bax ↑Bcl2; ↓P-P38 | [Yang *et al.*, 2016] | |
| CCl_4_-induced Liver injury | Luteolin | Male Wistar rats | -- | 100mg/kg | ↑nuclear nrf2, Ho-1, GSH-Px, SOD, CAT ↓MDA; ↓IL-6, IL-1β, and TNF-α  ↓Bax, caspase-3 ↑Bcl-2 | [Yan *et al.*, 2019] | |
| AFB1-induced  Liver injury | Luteolin | male C57BL/6 mice | -- | 50mg/kg | ↑nrf2, Ho-1, NQO1, GCLC, T-AOC, T-SOD, GSH-Px, CAT ↓ROS, MDA;  ↑Bcl-2 ↓Bax, caspase-3, cytochrome-c | [Rajput *et al.*, 2021] | |
| IMI-induced  liver fibrosis | luteolin | male Japanese quails | -- | 800mg/kg | ↑nuclear nrf2, Ho-1, NQO1, GSH, SOD ↓MDA; ↓p-NF-ΚB, TNF-𝛼, IL-1β, IL-6; ↓fibrosis-related genes | [Deng *et al.*, 2022] | |
| HgCl_2_-induced renal injury | luteolin | male Wistar rats | -- | 80mg/kg | ↑nuclear nrf2, Ho-1, NQO1, GSH ↓MDA; ↓nuclear NF-κB, TNF-α;  ↓Bax, P53 ↑Bcl-2, Bc-xL | [Tan *et al.*, 2018] |  |
| lead acetate-induced renal injury | luteolin | male Wistar albino rats | -- | 50mg/kg | ↑nrf2, Ho-1, SOD, CAT, GPX, GR, GSH ↓MDA; ↓IL-1𝛃, TNF-α, Nos2 and NO; ↓caspase 3, Bax ↑Bcl-2 | [Albarakati *et al.*, 2020] |  |
| Pb-induced  testicular injury | luteolin | Male Wistar albino rats | -- | 50mg/kg | ↑nrf2, Ho-1, GR, GPx, SOD, CAT ↓LPO, NO; ↓TNF-α, IL-1β, Nos2; ↓Bax, caspase-3 ↑Bcl-2 | [Al-Megrin *et al.*, 2020] | |
| Cadmium-induced  Testis Injury | wogonin | male Wistar rats | -- | 10mg/kg | ↓Keap1 ↑nrf2, NQO1, Ho-1, SOD, CAT, GPx ↓MDA; ↓p-IKKβ, p65, TNF-α ↑IL-10; ↓caspase-3, caspase-9, Bax | [Yu *et al.*, 2020] |  |

# Supplementary Table 2: Flavones activate Nrf2 pathways against various toxicants.
